# Investigating Correlation between Exercise Participation Motivation and Social Physique Anxiety and Their Differences across Exercise Stages of Change

**DOI:** 10.3390/sports12090239

**Published:** 2024-08-31

**Authors:** Erdal Macila, Erman Dogan, Nuriye Sancar

**Affiliations:** 1 Physical Education and Sports Department, Girne American University, North Cyprus via Mersin 10, 99320 Kyrenia, Turkey; erdalmacila_06@hotmail.com (E.M.); ermandogan@gau.edu.tr (E.D.); 2 Department of Mathematics, Near East University, North Cyprus via Mersin 10, 99138 Nicosia, Turkey

**Keywords:** behavioral regulations in exercise, social physique anxiety, exercise stages of change

## Abstract

This study aims to investigate the correlation between exercise participation motivation and social physique anxiety and their differences across the exercise stages of change in individuals. A convenience sampling technique was used to gather data from 374 sedentary individuals aged 18 and over, using a questionnaire that included the Behavioral Regulations in Exercise Questionnaire-2 (BREQ-2), Social Physique Anxiety (SPA) scale, and Exercise Stages of Change Questionnaire (ESOCQ). Welch’s ANOVA test was utilized to examine if there were significant differences between the average levels of behavioral regulations in exercise and SPA within exercise stages of change, respectively. Based on Welch’s ANOVA results, it has been found that there are significant differences in the total score of SPAS according to exercise stages of change (F = 15.801, *p* < 0.001). Furthermore, the sub-factors intrinsic regulation, introjected regulation, and external motivation statistically significantly differ according to exercise stages of change (F=6.681, *p* < 0.001 for IR; F=30.186, *p* < 0.001 for introjected regulation; F=10.104, *p* < 0.001 for external motivation, F=0.481, *p* = 0.750 for AM). Pearson’s r correlation coefficients were also computed to determine the relationship between behavioral regulations in exercise and SPA. A significant negative moderate correlation was found between intrinsic regulation and SPA (r = −0.645, *p* < 0.001). Furthermore, a significant medium positive correlation was found between introjected regulation and SPA (r = 0.534, *p* < 0.001), external motivation and SPA (r = 0.588, *p* < 0.001), and amotivation and SPA (r = 0.564, *p* < 0.001). The findings suggest that being self-motivated for exercise might decrease SPA. On the other hand, exercise motivated by external pressures could lead to increased SPA. The findings also suggest that those who started exercising with intrinsic motivation reached more advanced stages of exercise than those who started exercising with extrinsic factors. Finally, extrinsic motives may help in the first stages of the exercise, but intrinsic motives are required to continue the exercise. Consequently, these findings may guide physical activity specialists, trainers, etc., to develop more effective strategies to motivate exercise participation by considering social physique anxiety among individuals.

## 1. Introduction

Although the human body is designed for movement and physical activity, exercise is often excluded from the daily routine [1]. As the human body continues to live without physical activities, some functional skills decline, leading to various diseases [2]. In addition to these, regular physical activity positively influences emotional and mental health [3]. It has been shown that regular physical activity improves mental health by reducing signs of anxiety and stress, helping to moderate emotions like anger and aggressiveness, and improving sleep quality [4]. Since physical activity stabilizes the individual’s health, physical structure, flexibility, and weight, it increases self-perception, self-esteem, and endorphins lead to positive mood [5,6,7,8]. Therefore, in several developed countries, promoting physical exercise has become a main objective of public health because of the numerous benefits associated with it [9]. Considering these positive effects of physical activity, it is vital to make exercise a sustainable lifestyle, and thus, understanding the exercise behavior of individuals is important. Increasing physical activity participation can help to reach these objectives [10]. In this context, it is essential to reveal the psychological phenomena that effectively ensure the continuity of physical activity behavior [11]. Recently, psychological factors and concepts related to physical activity have been the researchers’ main concerns in sports and exercise psychology [12]. Studies in exercise and sports psychology indicate that participation in physical activity should be continuous to realize physical and psychological benefits [13]. Although the advantages of physical activity are well known, individuals who start exercising stop exercising after a short period [14]. To support a physically active lifestyle and increase the number of people who exercise regularly, it is important to identify the psychological and social aspects of physical activity that are beneficial. Discontinuation of exercise programs is partially based on motivation, which forms the basis of exercise behavior [15].

It is known that understanding changes in exercise behavior is a complex process that includes psychological, social, and environmental aspects [16]. Therefore, many theories have been developed to understand exercise behavior to understand better how people acquire and maintain exercise routines throughout life. When examining these theories, we notice that they address the sources of motivation that enable participation in exercise. According to Bandura’s [16] Social Cognitive Theory (SCT), an individual’s exercise behavior is shaped by their self-efficacy, which indicates their belief in their ability to engage in physical activity successfully. In addition, Bandura [16] suggested that individuals are motivated to exercise by the potential outcomes and rewards it offers. SCT proposes some interventions that can influence exercise behavior by focusing on increasing individuals’ self-efficacy and providing positive incentives for participation in physical activity [17]. When examining more recent theories of exercise behavior, we find that they discuss the importance of exercise motivation and the emotions that influence this motivation. The most important one, the Self-Determination Theory (SDT), highlights the role of motivation in transforming exercise behavior [18]. This theory also mentions some emotional states that provide this motivation. SDT highlights that people are more inclined to engage in physical activity when they experience a sense of autonomy, competence, and commitment [19]. The theory also suggests that these emotions are involved in developing intrinsic motivation [20]. According to SDT, intrinsic motivation is highlighted as significant, involving engaging in exercise for its own sake, as opposed to extrinsic motivation, such as rewards or external pressure [21]. In this context, the theory suggests some interventions to trigger intrinsic motivation. The theory suggests that these interventions should be based on emotions, claiming they are related to intrinsic motivation [19]. Theories that link changes in exercise behavior with motivation offer a new perspective for future research on their relationship.

The transtheoretical model is a stage theory, which presupposes that people go through several discrete “stages of change” on their path to acquiring and preserving, while numerous social-cognitive theories are thought of as continuum models of behavior [22]. The stages of change comprise five steps ranging from pre-contemplation, in which a person has not thought about changing their behavior, to maintenance, in which a person has effectively embraced a new behavior for at least six months and attempts to avoid reverting to old patterns of behavior [23]. In rare cases, the stages are extended to six or more. Although the transtheoretical model assumes that cognitive, emotional, and behavioral ‘change processes’ facilitate stage transitions, according to Prochaska and Velicer [24], there is some indication to suggest that these apply to other behaviors more so than to physical activity [25]. In this case, it would be more beneficial to examine the research on the examination of behavioral changes of individuals in our research design in terms of exercise. Marcus et al. [26] created measures to assess exercise self-efficacy as well as stages and methods of change in exercise. Moreover, they showed that the transtheoretical model may be utilized effectively in exercise behavior research by conducting studies on the development of measurement and model testing [27]. From this point of view, we can use the exercise stages of change theory, which deals with the behavioral changes of individuals in terms of exercise, in research involving processes related to physical activity.

Today, despite recognizing the positive influences of physical activity on mental health, many people find it challenging to stay motivated and consistent with their exercise routines. Some psychological factors contribute to this lack of consistency [28]. Therefore, it may indirectly have negative effects on mental health [29]. Exercise motivation encompasses the internal and external factors driving a person to participate in physical activity regularly [30]. Accordingly, it is crucial to identify which types of motivation are more correlated to mental health. Intrinsic and extrinsic motivation are the types of motivation that are thought to have the most impact on individuals’ mental health [28]. External motivation comes from rewards or competition, while intrinsic motivation comes from pleasure and satisfaction [28]. Individuals’ level and type of motivation can significantly influence their willingness to do physical activity [31]. Furthermore, the provision of need support (i.e., autonomy, competence, and relatedness support) and the avoidance of need thwarting are also essential factors in shaping one’s experience of motivation [32]. The classification of the concepts that make up this exercise motivation is discussed as behavioral regulations in exercise. Reviewing past research on behavioral regulations in exercise will help to assimilate the concept better. Past findings suggest that exercise intention positively relates to intrinsic motivation and introjected and identified regulation [33]. Furthermore, although some mixed correlational findings have been stated by Werman-Josefsson et al. [34], intrinsic motivation, integrated regulation, and identified regulation have generally been associated with increased physical activity [35,36,37]. Ultimately, a positive inverse link between the development of exercise identity and intrinsic motivation, recognized regulation, and introjected regulation has been observed [38]. In light of these studies, it is observed that the relationship between behavioral regulation and motivation in exercise. Therefore, examining the factors influencing exercise motivation may provide insights into change and regulation in exercise behavior.

It is essential to comprehend the motivation for engaging in physical activity and the psychological factors related to this concept, such as the Social Physique Anxiety (SPA) scale. This understanding is crucial when considering the role of this lifestyle choice in preventing disease and improving life quality [39]. Theoretical foundations of SPA are found in the literature on body image and body esteem and overcome the gap between a person’s perception of their body and their level of contentment or discontent with that image [40]. Body image is a multifaceted concept of perceptual, cognitive, emotional, and behavioral [41]. SPA also reveals an interpersonal aspect of body esteem and self-presentation concerning people’s feelings about how other people see their bodies [42]. Self-presentation, in particular, is a complicated process that involves the person’s desire to make a good impression and the perception of creating a desirable one for others [43]. These ‘desirable’ perceptions, especially in Western societies, frequently center on physical characteristics, such as an attractive look, a thin and toned body shape for women, and a muscular toned body for men [44]. It’s a case that is frequently made when studies discover a relationship between SPA and physical activity. SPA is generally an indicator of self-presentation processes or body image effects [45,46]. Sport and exercise psychology does not have a particular conceptual or theoretical model of SPA experiences, except theoretical origins in self-presentation, social anxiety, and body esteem studies. However, research on SPA has examined personality, self-esteem, motivation, social interactions, and emotion [42]. From the perspective of our research design, the relationship between SPA and motivation is crucial. SPA is one of the most prominent psychological factors correlated with motivation to participate in physical activity [47]. SPA is defined as the anxiety and tension people feel when others evaluate their physical appearance as one of the concepts related to the individual’s concerns about their appearance [40]. A significant contributing factor to social physique anxiety (SPA) is the pressure to meet societal expectations regarding physical appearance [48]. Environmental factors also play a role, including body image dissatisfaction and social comparison, which further increase SPA [49]. As a result, increased SPA can affect participation in physical activities and the selection of physical activities [47]. People with high levels of SPA can avoid physical activity, which can lead to decreased social interaction and potentially have negative effects on self-esteem [42]. These interactions may offer insight into the extent of the correlation between SPA and physical activity. Therefore, it is crucial to consider SPA in physical activity settings and to link it to physical activity levels and processes [50]. This link might help better understand the relationship that could impede the continuity of physical activity.

In light of the theories and research mentioned earlier, we can infer that motivation for exercise participation and SPA may influence individuals’ regulation and implementation of exercise behaviors. However, the continuity dimension of these concepts is also a significant issue that requires attention. Exercise behavior regulations are important for individuals to make their exercise habits continuous. At the same time, due to the long-term effects of SPA on exercise behaviors, reducing SPA may increase individuals’ adherence to exercise programs. Therefore, finding results on the relationship between SPA and motivation for exercise participation may be essential. Moreover, examining these two factors, which are assumed to be related to individuals’ exercise participation, in terms of exercise behaviors may offer a different perspective. From this point of view, this research aims to:

i. To examine the relationship between behavioral regulations in exercise and social physique anxiety
ii. To examine how social physique anxiety and behavioral regulations differ according to the stages of change during the exercise process, respectively

The research hypotheses created are listed below:

**H_1_**. *There is a significant negative correlation between social physique anxiety and intrinsic regulation*.

**H_2_**. *There is a significant positive correlation between social physique anxiety and introjected regulation*.

**H_3_**. *There is a significant positive correlation between social physique anxiety and external motivation*.

**H_4_**. *There is a significant positive correlation between social physique anxiety and amotivation*.

**H_5_**. *Social physique anxiety differs according to Exercise Stages of Change*.

**H_6_**. *Intrinsic regulation differs according to Exercise Stages of Change*.

**H_7_**. *Introjected regulation differs according to Exercise Stages of Change*.

**H_8_**. *Amotivation differs according to Exercise Stages of Change*.

**H_9_**. *External motivation differs according to Exercise Stages of Change*.

## 2. Materials and Methods

### 2.1. Study Design

This study was planned as cross-sectional quantitative research with the aim of examining the relationship between behavioral regulations in exercise and social physique anxiety and examining how social-physical anxiety and behavioral regulations differ according to the stages of change during the exercise process, respectively. Before starting this research, ethical approval was acquired from the Scientific Research Ethics Committee of Girne American University (23-24/6) dated 26 February 2024. This study was conducted in Nicosia and Kyrenia, two big cities in North Cyprus, between 1 March 2024 and 1 April 2024, after the ethical approval.

### 2.2. Population and Sample

The sedentary individuals over 18 who exercise as a leisure activity in university and private sports facilities in Northern Cyprus were determined as the target population. The inclusion criteria have been determined as sedentary individuals over the age of 18 who exercise as a leisure activity in university and private sports facilities, those who exercise at least twice a week, and those who do not have serious health problems that prevent them from exercising. The exclusion criteria were those who did not meet these criteria, in other words, persons under the age of 18 with serious health problems. In this study, the sedentary individual is defined as an individual who does not do sports professionally but does sports for leisure time. The participants’ answers to the questions in the personal information form about how many days and hours a week they participated in physical activity determined whether they had a sedentary lifestyle. Convenience sampling was used to gather data. A total of 450 people were invited, but 19 people refused to participate. Of the remaining 431 people, 57 people were not included in the study because they did not meet the inclusion criteria. Thus, 374 individuals who satisfied the inclusion criteria were included in the study. Data were collected from three universities and seven private sports facilities. The dataset has no missing observations and no outliers. A power analysis was performed using G*Power version 3.1.9.7 to identify the minimal sample size required to test the research hypothesis [51]. A minimum sample of n = 138 (medium effect size, 95% power) is necessary for correlation analysis, according to G-power results. Similarly, a minimum sample of n = 305 is necessary for ANOVAs (5 groups, medium effect size, 95% power, fixed effect, omnibus test, one way). This indicates that the sample size (n = 374) was adequate for this study.

### 2.3. Procedure

In the study, three scales, namely the Social Physique Anxiety (SPA) scale, Behavioral Regulations in Exercise Questionnaire-2 (BREQ-2), and Exercise Stages of Change Questionnaire (ESOCQ), were used. Additionally, demographic information about the participants was collected using the information form. In this information form are questions about age, gender, educational status, marital situation, weight, height, frequency of training, type of physical activity, and how long they have been training. The questionnaires were administered to the participants face to face. The paper-and-pencil survey was used in this study. Participants were to complete the surveys on-site. The purpose of the study was explained to the participants, and the necessary instructions were given to complete the questionnaires. Also, the questionnaires were presented in a quiet environment where the participants could easily answer them. Informed consent was acquired from all participants in the study. All responses were collected anonymously. Participants’ confidentiality and data security were meticulously ensured, with all completed surveys securely collected and stored for analysis.

### 2.4. Measurements

#### 2.4.1. Behavioral Regulations in Exercise Questionnaire-2 (BREQ-2)

The Behavioral Regulations in Exercise Questionnaire-2 (BREQ-2) was introduced by Mullen et al. [52] and revised by Markland and Tobin [53]. The BREQ-2 consists of 19 items and 4 subfactors. The BREQ-2 measures different exercise motivation types: external regulation, introjected regulation, intrinsic regulation, and amotivation. The intrinsic regulation subscale has seven items, while the external regulation, introjected regulation, and amotivation subscales consist of 4 items each. The BREQ-2 is a 5-point Likert-type questionnaire. The Turkish adaptation of the questionnaire was made by Ersöz et al. [54].

#### 2.4.2. Social Physical Anxiety (SPA) Scale

The Social Physique Anxiety (SPA) scale is a psychological assessment tool comprising 12 self-report items. It was introduced by Hart et al. [40] to measure Social Physique Anxiety (SPA). SPA has two subfactors: one measures feelings of discomfort, and the other evaluates expectations of negative evaluation. Participants answered on a 5-point Likert-type scale: not at all (1), slightly (2), moderately (3), very (4), and extremely (5). The total score of the scale can vary from 12 to 60, with higher values indicating more significant anxiety. The Turkish adaptation of the SPA scale was developed by Ballı and Aşçı [55].

#### 2.4.3. Exercise Stages of Change Questionnaire (ESOCQ)

The Exercise Stages of Change Questionnaire (ESOCQ) determined the person’s exercise behavior step. ESOCQ was developed by Marcus and Lewis [56], and Cengiz et al. [57] conducted its adaptation into Turkish. The questionnaire consists of 4 items. The items in the questionnaire, which tries to determine the participants’ willingness to exercise, are answered as yes/no. The questionnaire aims to determine five different but cyclical steps of exercise behavior change. These stages of change are precontemplation, contemplation, preparation, action, and maintenance. In the precontemplation stage, the person is inactive and does not plan to be active in the next six months. In the contemplation stage, the person is not physically active but intends to be active in the next six months. Another step is preparation, in which the person is physically active, but their physical activity level needs to be at the desired and recommended level. People in the action and maintenance stages have been physically active for less than or more than six months.

### 2.5. Data Analysis

R Studio version 4.3.2 was utilized for the statistical analyses. For descriptive statistics of demographic characteristics, continuous variables were described by mean with standard deviation and median with interquartile range values, and categorical variables were described with frequency and percentage values. Also, mean with standard deviation and median with interquartile range values, and maximum-minimum values of scale scores were calculated to determine participants’ behavioral regulations in exercise, SPA, and exercise stages of change, and Cronbach’s alpha values for scale and subscale have been evaluated for internal consistency measures of scales. According to the Levene homogeneity test results, the variances for total scale scores and subscale scores did not satisfy the homogeneity assumption. Welch’s ANOVA was chosen to compare the means of groups because Levene’s test resulted in the assumption that the homogeneity of variances was not met. Welch’s ANOVA is robust against violations of this assumption and provides more reliable results when variances are unequal. Similarly, the Games-Howell post hoc test was used, which does not require equal variances and is suitable for comparing group means under these conditions. Therefore, Welch’s ANOVA test was utilized to test significant differences between the average levels of behavioral regulations in exercise and SPA within exercise stages of change, respectively, and eta squared ($\eta^{2}$) calculated for the effect size for Welch’s ANOVA test. Since the variances were unequal, the Games-Howell test from the post hoc tests was employed. In addition, Pearson’s r coefficients were computed to test the association between behavioral regulations in exercise and SPA. Furthermore, to examine the impact of SPA on each subscale of the behavioral regulations in the exercise scale, a simple linear regression model was constructed. Before constructing simple linear regression models, there are many assumptions, such as linear correlation between dependent and independent variables, homoscedasticity (constant variance of residuals), no autocorrelation (independence of residuals), and residuals normality. The linearity assumption has been checked by scatterplots for X vs. Y. The normality assumption has been checked by the Kolmogorov-Smirnov test because the sample size is greater than 50. It can be concluded that the residuals are normally distributed if the *p* > 0.05 in the Kolmogorov-Smirnov test. Additionally, the homoscedasticity assumption was tested using the Breusch-Pagan test. Breusch-Pagan test is confirmed homoscedasticity when *p*-value > 0.05. Lastly, the Durbin-Watson statistic is utilized to test autocorrelations. The Durbin-Watson statistic between 1.5 and 2.5 suggests no autocorrelation [58].

## 3. Results

Table 1 shows the demographic information of participants. 58.6% (n = 219) of the participants were female, and 41.4% (n = 155) were male. The age of the participants in this sample varied between 18 and 59 years, with a mean age of 32.05 and a standard deviation of 9.02 (mean age for male: 30.30 with a standard deviation of 8.99; mean age for male: 33.29 with a standard deviation of 8.86). On the other hand, when the frequency of weekly physical activity was examined, the median number of physical activities per week was computed as 5 with an interquartile range of 1. It was observed that the most common physical activity frequency among participants was five times a week (55.6%, n = 208). The age of the participants in this sample varied between 18 and 59 years, with a mean age of 32.05 and a standard deviation of 9.02. Lastly, when examining how long the participants had been exercising, the duration ranged from 1 to 40 years (median = 6, interquartile range = 4), with an average physical activity duration of 8.55 years and a standard deviation of 6.44. Based on these results, most participants had been exercising for a long time. When exercise stages of change were investigated, the number of participants in the maintenance stage was relatively high compared to other stages (n = 282, 75.4%). This indicated that the participants were maintaining regular exercise habits. The number of participants in other stages was lower. The stage with the fewest participants was the pre-contemplation stage, where individuals did not think about or were inactive in physical activity (n = 12, 3.2%). Furthermore, the frequency distribution of physical activity types among participants has been presented in Table 2.

Additionally, descriptive statistics of the scales SPA and BREQ-2 have been presented in Table 3. The Cronbach’s alpha values indicated that the items within the scales were consistent with each other and that the scales were reliable. Here, the Cronbach’s alpha values of the scales were generally above 0.70, presenting a reasonable level of internal consistency. In addition to the table, the minimum and maximum values of all scales were 1 and 5, respectively.

**Figure 1:**
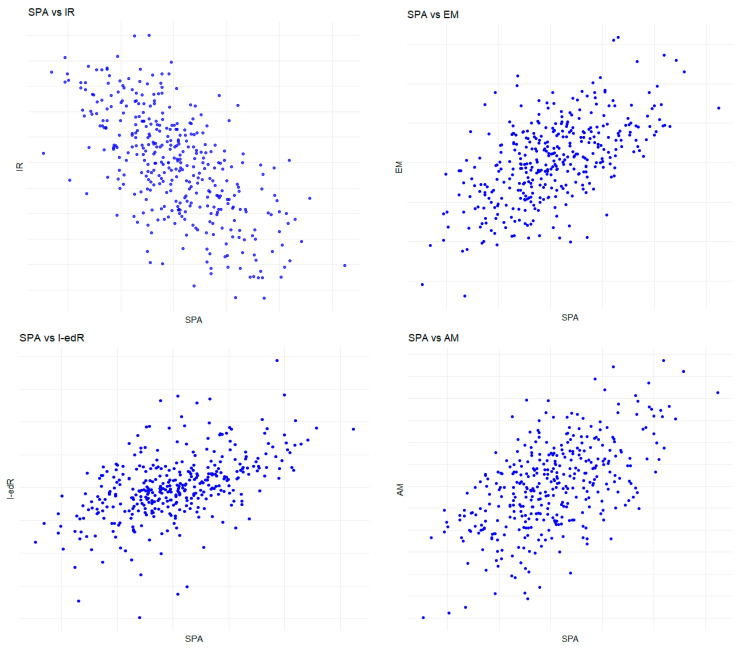
Scatter plots for the relationships between SPA and IR, I-edR, EM, and AM, respectively.

Before constructing simple linear models, all assumptions have been checked. The scatter plots of the SPA (independent variable) against IR, I-edR, EM, and AM (dependent variables for each model) have linear relationships, as shown in Figure 1, respectively. Additionally, according to Kolmogorov-Smirnov test for each model, it has been observed that the normal distribution of residuals has been verified since *p* > 0.05 for each model (D = 0.050677, *p* = 0.292 for SPA → IR; D = 0.061642, *p* = 0.117 for SPA → I-edR; D = 0.056046, *p*-value = 0.191 for SPA → EM; D = 0.064769, *p* = 0.0867 for SPA → AM). On the other hand, the Breusch-Pagan test has confirmed constant variance of residuals since each *p*-value is greater than 0.05 (χ^2^ = 2.587, *p* = 0.108 for SPA → IR; χ^2^ = 1.488, *p* = 0.223 for SPA → I-edR; χ^2^ = 1.239, *p* = 0.266 for SPA → EM; χ^2^ = 2.117, *p* = 0.146 for SPA → AM). Lastly, the Durbin-Watson statistic for each model has been calculated as DW = 1.867, DW = 1.631, DW = 1.824, and DW = 1.966, respectively, suggesting residuals were independent, and there was no autocorrelation for each simple linear model.

The following results were obtained when examining the correlations between behavioral regulations in exercise sub-dimensions and SPA, as seen in Table 4 and Table 5. The Pearson correlation coefficient of −0.645 indicates a significant negative moderate association was determined between intrinsic regulation and SPA, and the simple linear regression results (B = −0.645, β = −0.645, R^2^ = 0.416, *p* < 0.001) show that SPA significantly predicts intrinsic regulation, explaining 41.6% of the variance in IR. Therefore, H_1_ was supported by the results. On the other hand, a significant medium positive correlation was found between introjected regulation and SPA (r = 0.534, *p* < 0.001), and the simple linear regression findings (B = 0.741, standardized β = 0.534, R^2^ = 0.286 *p* < 0.001) show that SPA significantly predicts introjected regulation, explaining 28.6% of the variance in introjected regulation. Also, a significant medium positive association was determined between external motivation and SPA (r = 0.588, *p* < 0.001), and regression results (B = 0.463, standardized β = 0.588, R^2^ = 0.345, *p* < 0.001) indicate that external motivation is significantly predicted by SPA and 34.5% of the variability in external motivation can be explained by SPA. Lastly, r = 0.564 has shown a significant positive moderate correlation was found between amotivation and SPA, and the simple linear regression results (B = 0.367, standardized β = 0.564, R^2^ = 0.318, *p* < 0.001) have shown that SPA significantly predicts amotivation which explains 31.8% of the variance in amotivation. Therefore, the hypotheses H_2_, H_3_, and H_4_ were supported by these results.

According to Levene’s homogeneity test, the assumption of homogeneity of variances was not met for each scale score (F(4, 369) = 11.284, *p* < 0.001 for IR; F(4, 369) = 9.284, *p* < 0.001 for I-edR; F(4, 369) = 13.176, *p* < 0.001 for EM; F(4, 369) = 7.458, *p* < 0.001 for AM; F(4, 369) = 6.987, *p* < 0.001 for SPA). Therefore, the Welch ANOVA test was chosen to compare the mean SPA and subscales of the BREQ-2 levels within exercise stages of change. Table 6 illustrates Welch’s ANOVA results according to exercise stages of change. Descriptive statistics of SPA and the subscales of the BREQ-2 according to exercise stages of change can be seen in Table 3. According to these results, the total scores of SPA and behavioral regulations in exercise scales differ significantly across the exercise stages of change categories (F = 15.801, *η*^2^ = 0.063, *p* < 0.001). The Games-Howell post hoc test further confirms these findings, showing that the SPA score of the action group (M = 2.896) is significantly higher than the scores of the contemplation (M = 2.024), preparation (M = 2.241), and maintenance (M = 2.247) groups (*p* < 0.001). Thus, H_5_ was supported by Welch’s ANOVA test.

When examining the subscales of the BREQ-2, it was found that all subscales, except for amotivation (AM), differ across the exercise stages of change categories ($F=6.681,\;\eta^{2}=$0.041, *p* < 0.001 for IR, $F=30.186,\eta^{2}=$0.144, *p* < 0.001 for I-edR, $F=10.104,\;\eta^{2}=$0.055, *p* < 0.001 for EM, $F=0.481,\eta^{2}=$0.008, *p* = 0.750 for AM). Therefore, the hypotheses H_6_, H_7_, and H_9_ were supported, but H_8_ was not supported by Welch’s ANOVA test. Various exercise stages of change pairs with significant differences, according to the Games-Howell post hoc test, are shown in Table 6.

## 4. Discussion

Results indicated significant correlations between exercise participation motivation and SPA. When the relationship between SPA and behavioral regulations in exercise subscales was investigated, a significant negative association was observed between SPA and intrinsic regulation. It was revealed that the SPA levels of exercising individuals increased, and the scores of intrinsic regulation decreased. On the other hand, there was a positive significant correlation between SPA and external regulation, introjected regulation, and amotivation. It was indicated that the SPA levels of exercising individuals increased, and the scores of external regulation, introjected regulation, and amotivation also increased. Based on Welch’s ANOVA results, it has been found that there are significant differences in the total score of the SPA according to exercise stages of change (*p* < 0.001). Furthermore, the sub-factors intrinsic regulation, introjected regulation, and external motivation statistically significantly differ according to exercise stages of change (*p* < 0.001).

Studies revealing motivational orientations in exercise with SPA support the positive correlation between SPA and external regulation, which was also revealed in this study. External regulation is one of the types of motivation associated with self-determination theory and is positively related to SPA [19]. Sulu et al. [59], in their research with individuals who exercise regularly, found that those with high SPA also had high extrinsic motivation. Individuals more likely to endorse extrinsic motives for exercise had higher SPA levels [60]. Ullrich-French et al. [61] examined the relationship between SPA and external regulation in the physical education course and found that SPA was positively related to external regulation. Sabiston et al. [62] also found that extrinsic motives for exercise, like weight and appearance, were associated with SPA. Abdollahi et al. [63] also showed that physical appearance perfectionism and SPA are positively related. Lastly, athletes with external motives, appearance, and weight management had more significant SPA [64]. The literature consistently shows that when individuals are motivated to exercise by external regulation, such as physical appearance, attractiveness, or weight loss, their SPA levels increase.

The increase in physical appearance may be due to individuals not enjoying exercise, not having fun during the activity, or feeling guilty about their physical appearance. In addition, the lack of motivation may increase exercise behavior because individuals are uncomfortable with the negative evaluation of their physical appearance by others [47]. Previous research has shown a negative correlation between SPA and intrinsic regulation, similar to this study. Sulu et al. [59] found that intrinsic regulation predicted social physique anxiety negatively. Results indicate a possible inverse correlation between body-related anxiety and intrinsic motivation [65]. Self-presentational reasons for exercise, such as physical attractiveness, weight control, and body tone, were negatively correlated with SPA [66]. Based on these relationships, we can conclude that SPA may negatively predict intrinsic motivation [67]. Results revealed that individuals who enjoy exercise because they find it beneficial have lower SPA levels. Therefore, when individuals find exercise beneficial or enjoy exercise, they may not care about their physical appearance.

Evaluating the positive relationship between SPA and amotivation from the perspective of SDT, which was revealed in our research results, is a method addressed in past research. SDT suggests that motivation is multidimensional, and amotivation is the absence of both intrinsic and extrinsic motivation and is defined by the perception that an activity is worthless or won’t produce the intended results [47]. Self-determined motivation can potentially make working out more enjoyable, minimize societal judgments, and allay worries about one’s appearance [21]. However, SPA can also predict motivational regulations over time [67]. Cox et al. [68] illustrated that SPA predicts amotivation positively. Accordingly, high SPA can predict amotivation to exercise or manage regulations [15].

Although the correlation between body image motives and motivation types for exercise is complicated, previous research revealed that weight control motives and physical appearance were positively related to introjected regulation [69]. Physical appearance and weight control motives are essential factors that constitute the sub-dimensions of SPA. Based on this, we can infer that there may be a positive relationship between SPA and introjected regulation. Markland and Ingledew [70] made the most crucial suggestion about this relationship by revealing that SPA has a robust positive relationship with introjected regulation. Other research has also obtained results supporting this correlation direction. Cox et al. [71] have stated that introjected regulation may have a significant impact on promoting physical activity. Sulu et al.’s [59] research indicated that in exercise circumstances, introjected regulation is favorably predicted by appearance and weight management goals. These findings can be interpreted as part of the restorative and positive nature of emotions such as guilt, which are strongly associated with introjected regulation [72]. These results indicate that internal pressure or guilt-driven exercise motivation may be associated with SPA.

Correlation analysis shows a significant increase in SPA levels during the action stage of the exercise behavior change compared to the contemplation, preparation, and maintenance stages. In other words, the SPA of the participants in the action sub-dimension is higher than the participants in the other sub-dimensions. This may indicate that individuals in the action stage are more concerned about their physique, feeling a higher level of SPA that would enable them to increase exercise. The results suggest that SPA does not hinder exercise, but on the contrary, it may be a driver to exercise more. In addition, it suggests that a decrease in SPA may not occur during exercise but rather when exercise is sustained. When individuals exercise sustainably, their SPA decreases [47]. This is because health and physical appearance change when exercise becomes sustainable. Another interesting result was that SPA did not decrease linearly as the exercise stages of change progressed. The inconsistency of previous research results regarding the correlation between physical activity and SPA may help us understand our research results. Sicilia et al. [67] claimed that there is a complex correlation between SPA and the tendency to be physically active. When we examine the research results on this complex relationship, although there are findings that SPA decreases as the level of physical activity increases [73,74,75], we also see findings that SPA is not affected by the level of physical activity [68,76,77] or SPA increases as physical activity increases [60,78]. These results about the correlation between SPA and physical activity level suggest that SPA is not as important in determining physical activity level as expected. The correlation between SPA and physical activity level is possibly not as strong as expected, possibly because individuals’ motives to be active for health purposes are more dominant than their concerns about how they look physically.

The results also indicated a statistically significant difference in the participants’ exercise participation motivations according to the exercise stages of change. The sub-dimensions of behavioral regulations in exercise, intrinsic regulation, introjected regulation, and external regulation were significantly differentiated in different exercise stages.

The fact that individuals who are physically active or persistent in exercise have high levels of intrinsic regulation supports the fundamental idea of SDT [47]. Based on SDT, a positive relationship exists between self-determined motivation and exercise stages of change [19]. Many studies also showed that self-determined motivation positively affects physical activity levels [79,80,81,82]. Likewise, the results indicating that individuals who behave self-determined in exercise are at the advanced stages of exercise have been supported by many [83,84]. According to these findings, it can be said that self-determined behaviors will increase the level of physical activity, while individuals who show non-self-determined behaviors will adopt a sedentary lifestyle; those in the early stages of the exercise have low intrinsic motivation, while those in the advanced stages have higher intrinsic motivation.

On the other hand, the averages of introjected regulation fluctuate according to the stages. Previous research has indicated that those who are physically active or who show maintenance in exercise have higher mean levels of introjected regulation [23,85]. Although our study results showed higher introjected regulation scores in the maintenance stage compared to the first stages of the exercise, this increase did not fully support the previous literature as it did not increase linearly between the stages.

Finally, the differences between the means of external regulation in different exercise stages revealed significant results. The means of external regulation increased linearly in the pre-contemplation, contemplation, preparation, and action stages of the exercise. However, it decreased in the maintenance stage, which was the last stage of the exercise. Previous studies have also found that external motivation positively affects the level of physical activity [86,87] and indicated that individuals who start exercising with external motivation have a more significant effect in the first stages of exercise [88,89]. Starting exercise is usually decided for external reasons such as health and physical appearance. Therefore, the preliminary exercise stages can show less self-determined or more extrinsic exercise behavior. When individuals internalize their activities during exercise, they are more inclined to stick to their exercise [47]. Exercising out of personal choice, deriving enjoyment, and feeling competent and skilled during the activity all contribute to sustaining a commitment to exercise and strengthening one’s attachment to it.

In light of all these results, it is also necessary to mention the theoretical and practical implications of the study. From a theoretical standpoint, intrinsic regulation based on SDT, one of the motivation theories, is essential in reaching the advanced stages of exercise [21]. Also, the negative correlation between SPA and autonomous motivation (intrinsic regulation) and the positive association between SPA and controlling motivation (external regulation, introjected regulation, and amotivation) supported the theoretical propositions about the correlation between motivation types and SPA [71]. Our findings support previous research on the complex changes in SPA across different exercise stages, but they do not allow us to draw theoretical conclusions. However, our results also indicated that SPA reached its peak during the action stage but started to decline again during the maintenance stage. Based on this, we recommend taking steps to ensure that exercise becomes continuous, as simply motivating individuals to exercise is not sufficient to reduce SPA. Another practical study result revealed that intrinsic regulation is a positive predictor of reaching advanced stages of exercise. Therefore, guidance should be provided to ensure that individuals have high intrinsic regulation to make physical activity continuous. Similarly, based on the results that intrinsic regulation is an important factor in reducing individuals’ SPA levels, developing and implementing methods to increase intrinsic regulation can be important for reducing individuals’ SPA levels.

This study has several limitations. Firstly, this study is limited to the participant group, which consists of sedentary individuals 18 years and older who exercise leisurely in university gyms and private sports facilities in Northern Cyprus (Nicosia and Kyrenia cities). Also, this study is limited to the convenience sampling method used and self-reported measures used in this study. Finally, some uncontrolled variables, such as the socioeconomic status of the participants and cultural and environmental factors, were not taken into account, and this may have affected the findings of the study. Another limitation is the lack of detailed information about the participants’ lifetime physical activity history, which may affect the results. In future studies, it is suggested that researchers use participants with a more homogeneous distribution in exercise stages of change. It is also suggested that more participants can be reached and specific age ranges measured separately by grouping them. On the other hand, future research can also build on a classification system of motivational behaviors and develop and test the effectiveness of an intervention program to enhance autonomous forms of motivation among participants.

## 5. Conclusions

The research results indicated a relationship between SPA and exercise motivation, which can be explained by looking at how SPA relates to two types of motivation: autonomous and controlling. The results showed that SPA is negatively associated with autonomous motivation (intrinsic regulation) and positively associated with controlling motivation (external regulation, introjected regulation, and amotivation). The first finding suggests that being self-motivated for exercise might decrease SPA. On the other hand, the second finding suggests that exercise motivated by external pressures could lead to increased SPA.

On the other hand, the study’s results showed that participants in the advanced stages of exercise had higher intrinsic motivation, suggesting that those who started exercising with intrinsic motivation reached more advanced stages of exercise than those who started exercising with extrinsic factors. Another result is the inconsistency of intrinsic regulation, showing a non-linear difference between exercise stages, possibly due to participants’ non-homogeneity in different exercise stages. Lastly, external regulation scores increased in all exercise stages from the beginning. They decreased in the last exercise stage, maintenance, revealing that extrinsic motives may help start the first stages of the exercise. However, intrinsic motives are required for the continuation of exercise. Finally, results indicated that SPA was higher in the action stage than in the previous stages but decreased again in the maintenance stage. This shows that exercising is not enough to decrease SPA, but SPA decreases when exercise becomes continuous. It is thought that this research will help understand the relationship between SPA and exercise motivation and their effects on different exercise stages. Also, these findings may guide physical activity specialists, trainers, etc., to develop more effective strategies to motivate exercise participation by considering social physique anxiety among individuals.

## Figures and Tables

**Table 1:** Demographic characteristics.

Variable		f	%
Gender	Female	219	58.6
	Male	155	41.4
Education level	Primary School	4	1.1
	Secondary School	2	0.5
	High school	54	14.4
	Undergraduate	248	66.3
	Graduate	66	17.7
Age groups	18–25	90	24.1
	26–29	102	27.3
	30–34	44	11.8
	35–39	50	13.4
	40–44	52	13.9
	45–49	26	7.0
	50–54	4	1.1
	55–59	6	1.6
Marital status	Single	131	35.0
	Married	46	12.3
	Divorced	105	28.1
	Others	92	24.6
Socio-economic status	Low	12	3.2
	Medium	235	62.8
	High	127	34.0
Number of physical activities per week *	2	6	1.6
	3	34	9.1
	4	66	17.6
	5	208	55.6
	6	56	15.0
	7	4	1.1
Exercise Stages of Change	Precontemplation (S1)	12	3.2
	Contemplation (S2)	28	7.5
	Preparation (S3)	28	7.5
	Action (S4)	24	6.4
	Maintenance (S5)	282	75.4

f: frequency * Average Duration per physical activity: 1 h.

**Table 2:** Frequency distribution of physical activity types among participants.

Type of Physical Activity	Cardio	Yoga/Pilates	Others	Only
Weight Training	136	15	8	68
Cardio	-	20	2	72
Yoga/Pilates	-	-	-	4
Others	-	-	-	5

**Table 3:** Descriptive statistics of the scales.

Scale/Subfactors		N	M	SD	SE	Median (IntQuarRange)
Behavioral Regulations in Exercise Questionnaire-2 (BREQ-2) Cronbach’s Alpha: 0.854	S1	12	2.544	0.432	0.125	2 (1)
	S2	28	2.665	0.355	0.067	2 (1)
	S3	28	3.041	0.458	0.087	3 (1)
	S4	24	2.377	0.194	0.039	2 (1)
	S5	282	2.809	0.356	0.021	2 (1)
	Total	374	2.779	0.383	0.020	2 (1)
Intrinsic Regulation (IR) Cronbach’s Alpha: 0.804	S1	12	3.857	1.041	0.300	4 (1)
	S2	28	4.082	0.755	0.143	4 (1)
	S3	28	4.133	0.761	0.144	4 (1)
	S4	24	3.809	0.410	0.084	4 (1)
	S5	282	4.285	0.683	0.041	4 (1)
	Total	374	4.214	0.706	0.037	4 (1)
Introjected Regulation (I-edR) Cronbach’s Alpha: 0.835	S1	12	2.917	1.134	0.328	3 (1)
	S2	28	2.857	1.030	0.195	3 (1)
	S3	28	4.142	1.212	0.229	4 (1)
	S4	24	2.229	0.410	0.084	3 (1)
	S5	282	3.213	0.885	0.053	3 (1)
	Total	374	3.183	0.979	0.051	3 (1)
External Motivation (EM) Cronbach’s Alpha: 0.798	S1	12	1.167	0.195	0.056	2 (1)
	S2	28	1.286	0.526	0.099	2 (1)
	S3	28	1.786	0.981	0.185	2 (1)
	S4	24	1.188	0.111	0.023	2 (1)
	S5	282	1.399	0.511	0.030	2 (1)
	Total	374	1.398	0.553	0.029	2 (1)
Amotivation (AM) Cronbach’s Alpha: 0.784	S1	12	1.250	0.584	0.169	2 (1)
	S2	28	1.375	0.567	0.107	2 (1)
	S3	28	1.286	0.526	0.099	2 (1)
	S4	24	1.208	0.569	0.116	2 (1)
	S5	282	1.231	0.424	0.025	2 (1)
	Total	374	1.245	0.459	0.024	2 (1)
Social Physique Anxiety Scale (SPA) Cronbach’s Alpha: 0.892	S1	12	2.472	1.156	0.333	2 (1)
	S2	28	2.024	0.590	0.112	2 (1)
	S3	28	2.241	0.529	0.099	2 (1)
	S4	24	2.896	0.378	0.077	2 (1)
	S5	282	2.247	0.703	0.042	2 (1)
	Total	374	2.279	0.705	0.036	2 (1)
Physical Appearance Comfort (PAC) Cronbach’s Alpha: 0.873	S1	12	2.733	1.330	0.384	3 (1)
	S2	28	2.529	0.520	0.098	3 (1)
	S3	28	2.807	0.509	0.096	3 (1)
	S4	24	3.300	0.685	0.140	3 (1)
	S5	282	2.539	0.846	0.050	3 (1)
	Total	374	2.613	0.835	0.043	3 (1)
Expectation of Negative Evaluation (ENE) Cronbach’s Alpha: 0.821	S1	12	2.286	1.23041	0.355	2 (1)
	S2	28	1.663	0.735	0.139	2 (1)
	S3	28	1.837	0.781	0.148	2 (1)
	S4	24	2.607	0.305	0.062	2 (1)
	S5	282	2.039	0.772	0.046	2 (1)
	Total	374	2.040	0.786	0.041	2 (1)

M: Mean; SD: Standard deviation; SE: Standard error; IntQuarRange: InterQuartile Range.

**Table 4:** Correlation analysis results.

Variables	SPA	IR	I-edR	EM	AM
SPA					
IR	−0.645 **				
I-edR	0.534 **	0.415 **			
EM	0.588 **	−0.292 **	−0.084		
AM	0.564 **	−0.628 **	−0.083	0.311 **	

** *p* < 0.001 (n = 374).

**Table 5:** Simple linear regression analysis results.

	B	Std. Error	β	T	*p*	95% CI
SPA → IR						
Constant	4.867	0.118		41.083	<0.001	(4.634, 5.100)
IR	−0.645	0.050	−0.645	−13.001	<0.001	(−0.742, −0.547)
Model 1 summary: R^2^ = 0.416; F(1, 372) = 263.086; *p* < 0.0001						
SPA → I-edR						
Constant	3.720	0.169		21.983	<0.001	(3.388, 4.053)
I-edR	0.741	0.071	0.534	10.649	<0.001	(0.602, 0.880)
Model 2 summary: R^2^ = 0.286; F(1, 372) = 149.623; *p* < 0.0001						
SPA → EM						
Constant	0.918	0.093		9.842	<0.001	(0.735, 1.102)
EM	0.463	0.039	0.588	11.903	<0.001	(0.387, 0.540)
Model 3 summary: R^2^ = 0.345; F(1, 372) = 196.381; *p* < 0.0001						
SPA → AM						
Constant	0.925	0.079		11.780	<0.001	(0.770, 1.079)
AM	0.367	0.033	0.564	11.264	<0.001	(0.302, 0.432)
Model 4 summary: R^2^ = 0.318; F(1, 372) = 174.142; *p* < 0.0001						

B: Unstandardized regression coefficient, β: Standardized regression coefficient, CI: Confidence Interval.

**Table 6:** Welch’s ANOVA results according to exercise stages of change.

	Welch’ F Statistic	df1	df2	*p*	Significant Difference
IR	6.681	4	43.265	<0.001	S4-S5
I-edR	30.186	4	44.303	<0.001	S3-S1, S2, S4, S5 S4-S2, S5
EM	10.104	4	51.119	<0.001	S1-S3, S5 S4-S3, S5
AM	0.481	4	41.133	0.750	-
SPA	15.801	4	44.647	0 < 0.001	S4-S2, S3, S5

df: degrees of freedom.

## Data Availability

The data that support the findings of this study are available on request from the corresponding author.

## References

[1] Haskell W.L. (1996). Physical Activity, Sport, and Health: Toward the Next Century. Res. Q. Exerc. Sport..

[2] Izquierdo M., Merchant R.A., Morley J.E., Anker S.D., Aprahamian I., Arai H., Aubertin-Leheudre M., Bernabei R., Cadore E.L., Cesari M. (2021). International exercise recommendations in older adults (ICFSR): Expert consensus guidelines. J. Nutr. Health Aging.

[3] Tamminen N., Reinikainen J., Appelqvist-Schmidlechner K., Borodulin K., Mäki-Opas T., Solin P. (2020). Associations of physical activity with positive mental health: A population-based study. Ment. Health Phys. Act..

[4] Parra E., Arone A., Amadori S., Mucci F., Palermo S., Marazziti D. (2020). Impact of physical exercise on psychological well-being and psychiatric disorders. J. ReAtt. Ther. Dev. Divers..

[5] Daley A.J. (2002). Exercise therapy and mental health in clinical populations: Is exercise therapy a worthwhile intervention?. Adv. Psychiatr. Treat..

[6] Dąbrowska-Galas M., Dąbrowska J. (2021). Physical activity level and self-esteem in middle-aged women. Int. J. Environ. Res. Public. Health.

[7] Powell L.D., Gill D.L., Reifsteck E.J., Brown P.K. (2022). A physical activity program to promote mental health. Recreat. Sports J..

[8] Rodrigues F., Faustino T., Santos A., Teixeira E., Cid L., Monteiro D. (2022). How does exercising make you feel? The associations between positive and negative affect, life satisfaction, self-esteem, and vitality. Int. J. Sport Exerc. Psychol..

[9] Sallis J.F., Adlakha D., Oyeyemi A., Salvo D. (2020). An international physical activity and public health research agenda to inform coronavirus disease-2019 policies and practices. J. Sport. Health Sci..

[10] Guthold R., Stevens G.A., Riley L.M., Bull F.C. (2020). Global trends in insufficient physical activity among adolescents: A pooled analysis of 298 population-based surveys with 16 million participants. Lancet Child. Adolesc. Health.

[11] Bácsné-Bába É., Ráthonyi G., Pfau C., Müller A., Szabados G.N., Harangi-Rákos M. (2021). Sustainability-sport-physical activity. Int. J. Environ. Res. Public. Health.

[12] Biddle S.J.H., Fox K.R., Boutcher S.H. (2000). Physical Activity and Psychological Well-Being.

[13] Bull F.C., Al-Ansari S.S., Biddle S., Borodulin K., Buman M.P., Cardon G., Carty C., Chaput J.P., Chastin S., Chou R. (2020). World Health Organization 2020 guidelines on physical activity and sedentary behavior. Br. J. Sports Med..

[14] DiPietro L., Al-Ansari S.S., Biddle S.J., Borodulin K., Bull F.C., Buman M.P., Cardon G., Carty C., Chaput J.P., Chastin S. (2020). Advancing the global physical activity agenda: Recommendations for future research by the 2020 WHO physical activity and sedentary behavior guidelines development group. Int. J. Behav. Nutr. Phys. Act..

[15] Ntoumani C.T., Ntoumanis N. (2006). The role of self-determined motivation in the understanding of exercise-related behaviours, cognitions and physical self-evaluations. J. Sports Sci..

[16] Bandura A. (1986). Social Foundations of Thought and Action.

[17] Schunk D.H., DiBenedetto M.K. (2020). Motivation and social cognitive theory. Contemp. Educ. Psychol..

[18] Beauchamp M.R., Crawford K.L., Jackson B. (2019). Social cognitive theory and physical activity: Mechanisms of behavior change, critique, and legacy. Psychol. Sport Exerc..

[19] Deci E.L., Ryan R.M. (2012). Self-determination theory. Handb. Theor. Soc. Social Psychol..

[20] Ryan R.M., Ryan W.S., DiDomenico S.I., Deci E.L., Ryan R.M. (2019). The nature and the conditions of human autonomy and flourishing: Self-determination theory and basic psychological needs. The Oxford Handbook of Human Motivation.

[21] Ryan R.M., Deci E.L. (2000). Self-determination theory and the facilitation of intrinsic motivation, social development, and well-being. Am. Psychol..

[22] Prochaska J.O., DiClemente C.C. (1986). Toward a comprehensive model of change. Treating Addictive Behaviors: Processes of Change.

[23] Mullan E., Markland D. (1997). Variations in self-determination across the stages of change for exercise in adults. Motiv. Emot..

[24] Prochaska J.O., Velicer W.F. (1997). The transtheoretical model of health behavior change. Am. J. Health Promot..

[25] Marshall S.J., Biddle S.J. (2001). The transtheoretical model of behavior change: A meta-analysis of applications to physical activity and exercise. Ann. Behav. Med..

[26] Marcus B.H., Selby V.C., Niaura R.S., Rossi J.S. (1992). Self-efficacy and the stages of exercise behavior change. Res. Q. Exerc. Sport.

[27] Marcus B.H., Rakowski W., Rossi J.S. (1992). Assessing motivational readiness and decision making for exercise. Psychologica.

[28] Bandura A. (2014). Exercise of personal agency through the self-efficacy mechanism. Self-Efficacy.

[29] Sheehan R.B., Herring M.P., Campbell M.J. (2018). Associations between motivation and mental health in sport: A test of the hierarchical model of intrinsic and extrinsic motivation. Front. Psychol..

[30] Stults-Kolehmainen M.A., Blacutt M., Bartholomew J.B., Gilson T.A., Ash G.I., McKee P.C., Sinha R. (2020). Motivation states for physical activity and sedentary behavior: Desire, urge, wanting, and craving. Front. Psychol..

[31] Ntoumanis N., Ng J.Y., Prestwich A., Quested E., Hancox J.E., Thøgersen-Ntoumani C., Deci E.L., Ryan R.M., Lonsdale C., Williams G.C. (2021). A meta-analysis of self-determination theory-informed intervention studies in the health domain: Effects on motivation, health behavior, physical, and psychological health. Health Psychol. Rev..

[32] Ahmadi A., Noetel M., Parker P., Ryan R.M., Ntoumanis N., Reeve J., Beauchamp M., Dicke T., Yeung A., Ahmadi M. (2023). A classification system for teachers’ motivational behaviors recommended in self-determination theory interventions. J. Educ. Psychol..

[33] Howard J.L., Bureau J.S., Guay F., Chong J.X., Ryan R.M. (2021). Student motivation and associated outcomes: A meta-analysis from self-determination theory. Perspect. Psychol. Sci..

[34] Weman-Josefsson K., Lindwall M., Ivarsson A. (2015). Need satisfaction, motivational regulations and exercise: Moderation and mediation effects. Int. J. Behav. Nutr. Phys. Act..

[35] Duncan L.R., Hall C.R., Wilson P.M., Jenny O. (2010). Exercise motivation: A cross-sectional analysis examining its relationships with frequency, intensity, and duration of exercise. Int. J. Behav. Nutr. Phys. Act..

[36] Owen K.B., Smith J., Lubans D.R., Ng J.Y., Lonsdale C. (2014). Self-determined motivation and physical activity in children and adolescents: A systematic review and meta-analysis. Prev. Med..

[37] Dishman R.K., McIver K.L., Dowda M., Pate R.R. (2018). Declining physical activity and motivation from middle school to high school. Med. Sci. Sports Exerc..

[38] Ntoumanis N., Stenling A., Thøgersen-Ntoumani C., Vlachopoulos S., Lindwall M., Gucciardi D.F., Tsakonitis C. (2018). Longitudinal associations between exercise identity and exercise motivation: A multilevel growth curve model approach. Scand. J. Med. Sci. Sports.

[39] Zartaloudi A., Christopoulos D., Kelesi M., Govina O., Mantzorou M., Adamakidou T., Karvouni L., Koutelekos I., Evangelou E., Fasoi G. (2023). Body Image, Social Physique Anxiety Levels and Self-Esteem among Adults Participating in Physical Activity Programs. Diseases.

[40] Hart E.A., Leary M.R., Rejeski W.J. (1989). Tie measurement of social physique anxiety. J. Sport Exerc. Psychol..

[41] Cash T., Smolak L. (2011). Body Image: A Handbook of Science, Practice, and Prevention.

[42] Sabiston C.M., Pila E., Pinsonnault-Bilodeau G., Cox A.E. (2014). Social physique anxiety experiences in physical activity: A comprehensive synthesis of research studies focused on measurement, theory, and predictors and outcomes. Int. Rev. Sport Exerc..

[43] Martin Ginis K.A., Leary M.R. (2004). Self-presentational processes in health-damaging behavior. J. Appl. Sport Psychol..

[44] Thompson A.M., Heinberg L.J., Altabe M., Tantleff-Dunn S. (1999). Exacting Beauty: Theory, Assessment, and Treatment of Body Image Disturbance.

[45] Martin Ginis K.A., Mack D.E., Roberts G.C., Treasure D.C. (2012). Understanding exercise behavior: A self-presentational persepctive. Advances in Motivation in Sport and Exercise.

[46] Bane S.M., McAuley E., Duda J.L. (1998). Body image and exercise. Advances in Sport and Exercise Psychology Measurement.

[47] Ersöz G. (2016). An examination of motivational regulations, dispositional flow and social physique anxiety among college students for exercise: A self-determination theory approach. Coll. Stud. J..

[48] Eklund R.C., Mack D., Hart E. (1996). Factorial validity of the social physique anxiety scale for females. J. Sport Exerc. Psychol..

[49] Koyuncu M., Tok S., Canpolat A.M., Catikkas F. (2010). Body image satisfaction and dissatisfaction, social physique anxiety, self-esteem, and body fat ratio in female exercisers and nonexercisers. SBP J..

[50] Tsartsapakis I., Zafeiroudi A., Vanna G., Gerou M. (2023). Relationships of body dissatisfaction and self-esteem with social physique anxiety among university students in different study programs. Trends Psychol..

[51] Faul F., Erdfelder E., Lang A.G., Buchner A. (2007). G*Power 3: A flexible statistical power analysis program for the social, behavioral, and biomedical sciences. Beha. Res. Methods.

[52] Mullen E., Markland D., Ingledew D.K. (1997). A graded conceptualization of self-determination in the regulation of exercise behavior: Development of a measure using confirmatory factor analysis procedures. Pers. Individ. Dif..

[53] Markland D., Tobin V. (2004). A modification of the Behavioral Regulation in Exercise Questionnaire to include an assessment of motivation. J. Sport Exerc. Psychol..

[54] Ersöz G., Aşçı F.H., Altıparmak E. (2012). Reliability and validity behavioral regulations in exercise questionnaire-2. Turkiye Klin. J. Sports Sci..

[55] Ballı Ö.M., Aşçı F.H. (2006). Reliabılity and validity of social physique anxiety scale. J. Sport Sci..

[56] Marcus B.H., Lewis B.A. (2003). Physical Activity and the Stages of Motivational Readiness for Change Model. Pres. Counc. Phys. Fit. Sports Res. Dig..

[57] Cengiz C., Aşçı F.H., İnce M.L. (2010). Exercise stages of change questionnaire: Its reliability and validity. Turk. Clin. J. Sports Sci..

[58] Del Águila M.R., Benítez-Parejo N. (2011). Simple linear and multivariate regression models. Allergol. Immunopathol..

[59] Sulu B., Çakaloğlu E., Koruç P.B. (2021). Exercise Motivation and Social Physique Anxiety ın Adults. Int. J. Sport Cult. Sci..

[60] Frederick C.M., Morrison C.S. (1996). Social physique anxiety: Personality constructs, motivations, exercise attitudes, and behaviors. Percept. Mot. Ski..

[61] Ullrich-French S., Cox A.E., Rhoades Cooper B. (2016). Examining combinations of social physique anxiety and motivation regulations using latent profile analysis. Meas. Phys. Educ. Exerc. Sci..

[62] Sabiston C.M., Crocker P.R., Munroe-Chandler K.J. (2005). Examining current-ideal discrepancy scores and exercise motivations as predictors of social physique anxiety in exercising females. J. Sport Behav..

[63] Abdollahi A., Prasad K.D.V., Abdelrasheed N.S.G., Alshahrani S.H., Shoja S.J., Al-Awsi G.R.L., Estrada-Araoz E.G., Singer N., Ramírez-Coronel A.A., Mustafa Y.F. (2023). An investigation of relationships between body compassion, social physique anxiety and physical appearance perfectionism in young people from Iran. J. Eat. Disord..

[64] Williams P.A., Cash T.F. (2001). Effects of a circuit weight training program on the body images of college students. Int. J. Eat. Disord..

[65] Erturan-Ilker G. (2014). Psychological well-being and motivation in a Turkish physical education context. Educ. Psychol. Pr. Pract..

[66] Eklund R.C., Crawford S. (1994). Active women, social physique anxiety, and exercise. J. Sport Exercise Psy..

[67] Sicilia Á., Sáenz-Alvarez P., González-Cutre D., Ferriz R. (2016). Social physique anxiety and intention to be physically active: A self-determination theory approach. Res. Q. Exerc. Sport.

[68] Cox A.E., Ullrich-French S., Madonia J., Witty K. (2011). Social physique anxiety in physical education: Social contextual factors and links to motivation and behavior. Psychol. Sport Exerc..

[69] Sicilia A., Sáenz-Alvarez P., González-Cutre D., Ferriz R. (2014). Exercise motivation and social physique anxiety in adolescents. Psychologica.

[70] Markland D., Ingledew D.K., Hagger M.S.N., Chatzisarantis L.D. (2007). Exercise participation motives: A self-determination theory perspective. Intrinsic Motivation and Self-Determination in Exercise and Sport.

[71] Cox A.E., Ullrich-French S., Sabiston C.M. (2013). Using motivation regulations in a person-centered approach to examine the link between social physique anxiety in physical education and physical activity-related outcomes in adolescents. Psychol. Sport Exerc..

[72] Sabiston C.M., Brunet J., Kowalski K.C., Wilson P.M., Mack D.E., Crocker P.R.E. (2010). The role of body-related self-conscious emotions in motivating women’s physical activity. J. Sport Exerc. Psychol..

[73] Kowalski N.P., Crocker P.R.E., Kowalski K.C. (2001). Physical self and physical activity relationships in college women: Does social physique anxiety moderate effects?. Res. Q. Exerc. Sport.

[74] Brunet J., Sabiston C.M. (2009). Social physique anxiety and physical activity: A self determination theory perspective. Psychol. Sport Exerc..

[75] Cumming J., Thøgersen-Ntoumani C. (2011). Self-presentational cognitions for exercise in female adolescents. J. Appl. Soc. Psychol..

[76] Crawford S., Eklund R.C. (1994). Social physique anxiety, reasons for exercise, and attitudes toward exercise settings. J. Sport Exerc. Psychol..

[77] Melbye L., Tenenbaum G., Eklund R. (2008). Self-objectification and exercise behaviors: The mediating role of social physique anxiety. J. Appl. Biobehav. Res..

[78] Hausenblas H.A., Brewer B.W., Van Raalte J.L. (2004). Self-presentation and exercise. J. Appl. Sport Psychol..

[79] Flintoff A., Scraton S. (2001). Stepping into active leisure? Young women’s perceptions of active lifestyles and their experiences of school physical education. Sport Educ. Soc..

[80] Wilson P.M., Rodgers W.M. (2004). The relationship between perceived autonomy support, exercise regulations and behavioral intentions in women. Psychol. Sport Exerc..

[81] Daley A.J., Duda J.L. (2006). Self-determination, stage of readiness to change for exercise, and frequency of physical activity in young people. Eur. J. Sport Sci..

[82] Edmunds J., Ntoumanis N., Duda J.L. (2006). A test of self-determination theory in the exercise domain. J. Appl. Soc. Psychol..

[83] Wilson P.M., Rodgers W.M. (2002). The Relationship Between Exercise Motives and Physical Self-Esteem in Female Exercise Participants: An Application of Self-Determination Theory 1. J. Appl. Biobehav. Res..

[84] Rose E.A., Parfitt G., Williams S. (2005). Exercise causality orientations, behavioural regulation for exercise and stage of change for exercise: Exploring their relationships. Psychol. Sport Exerc..

[85] Chatzisarantis N.L., Hagger M.S., Biddle S.J., Smith B., Wang J.C. (2003). A meta-analysis of perceived locus of causality in exercise, sport, and physical education contexts. J. Sport Exerc. Psychol..

[86] Alexandris K., Tsorbatzoudis C., Grouios G. (2002). Perceived constraints on recreational sport participation: Investigating their relationship with intrinsic motivation, extrinsic motivation and amotivation. J. Leis. Res..

[87] Porter S. (2002). Physical Activity: An Exploration of the İssues and Attitudes of Teenage Girls.

[88] Landry J.B., Solmon M.A. (2004). African American women’s self-determination across the stages of change for exercise. J. Sport Exerc. Psychol..

[89] Wininger S.R. (2007). Self-determination theory and exercise behavior: An examination of the psychometric properties of the exercise motivation scale. J. Appl. Sport Psychol..

